# The Works of William Hewson, F.R.S.

**Published:** 1847-01-01

**Authors:** 


### MEDICO-CHIRURGICAL

### REVIEW.

JANUARY, 1847.

I. The Works of William Hewson, F.R.S. Edited, with an Introduction and Notes, by *George Gulliver*, F.R.S. London, printed for the Sydenham Society, 1846.

II. The Blood-corpuscle considered in its different Phases of Development in the Animal Series. Memoirs 1, 2, and 3. By *J. Wharton Jones*, F.R.S. Philosophical Transactions, Part 2, 1846.

We rejoice to find that the researches of one of the most distinguished physiologists of the English school, have been published in a collective form; and have thus been rescued from the kind of neglect in which they have for so long a period been allowed to remain. For this act of posthumous justice, the best acknowledgments of all who are interested, no less in the claims of merit than in the diffusion of sound scientific knowledge, are due to the Council of the Sydenham Society, who have, by this and other selections, evinced a just appreciation of the leading objects for the promotion of which this flourishing institution was established. It was an act peculiarly becoming such an Association, at an epoch when so many kindred minds have, in all parts of Europe, given a new and philosophic direction to the science of organization, to vindicate the claims of our countryman as a fellow-labourer in the same field, and as an original observer of the highest order. Owing to causes which it is difficult entirely to explain, but among the most influential of which may be placed the early death of Hewson, and the blaze which surrounded the two Hunters, the full value of the researches of this most sagacious investigator, not merely as regards the properties of the blood, touching which only he is known to the majority of readers, but as bearing upon some of the highest problems in physiology, has never yet been properly recognised. The satisfaction we have just expressed, is considerably heightened by perceiving that the editor, to whose charge these "Works" have been committed, has entered upon his duties with a jealons care of the reputation of their author; allowing no occasion where the claims of Hewson demanded vindication, to pass by unheeded; and never omitting to proclaim how frequently he had entered upon those paths, which, followed out in subsequent times, have conducted more than one observer to the highest distinction in the temple of science.

The cautious, minute, and accurate experiments upon the changes induced in the red corpuscles by saline solutions, by water and other fluids, to which we shall subsequently revert, and which appeared so little promising of great results at the time when they were made, might, had Hewson's valuable life been spared, have led to the discovery of the endosmotic powers of organic membrane, ultimately revealed by the admirable researches of Dutrochet, and to which the phenomena just noticed are so closely allied.

> "His researches concerning the use of the glands without regular excretory ducts show the marks of an active and ingenious mind, and I believe first indicated a rational method of research into this important and recondite branch of physiology. His observations on the lymphatic glands are in the same spirit; and his doctrine of central particles, so long looked upon as visionary, was a step far in advance of the age in which he lived." P. xlvii.

Those who are well acquainted with the full scope of Hewson's inquiries, will recognise the justice of the following passage, in which Mr. Gulliver, in alluding to his early death, and to that of his talented and honest friend, Magnus Falconar, observes, "had these two ingenious men lived to continue the inquiry which they had so successfully begun, it is not improbable that the important results obtained by Schwann would long since have been anticipated in this country; for, as I have elsewhere remarked, the researches of the German physiologist show that what Hewson propounded of the blood-corpuscles particularly is applicable to the tissues generally, and that the cell-nucleus of the present day is but another term for the central particle of Hewson." P. xlviii.

If to these instances it be added that Hewson was the first to determine the true figure of the red corpuscle; the first to examine accurately the fluid of the lymphatic system and of the thymus gland; the first distinctly to describe the pale corpuscles or lymph globules, as they are often called, of the blood; to say nothing of the many important facts he discovered respecting the lymphatic vessels, the fibrin and red corpuscles in the process of coagulation, and other valuable researches which it is unnecessary further to specify; if, we say, all this was accomplished by one person who lived only to his 35th year, enough has been adduced to justify the high estimation in which it is clear Hewson was held by his contemporaries, and by the limited number of modern anatomists who were, up to the present time, thoroughly acquainted with his labours.

The biography of the good and great, with whom our author may justly be classed, is one of the most delightful and instructive departments of literature; and among the other obligations we owe Mr. Gulliver for this admirable volume, not the least is the gratification he has afforded us by the interesting sketch he has prefixed of Hewson's life, mingled as it is with allusions to several of his intimate friends and contemporaries, foremost among whom stands the illustrious Franklin. It is a touching incident that the materials for this sketch were collected by the affectionate care of Mrs. Hewson, a woman of superior intelligence, to whom, indeed, Dr. Franklin addressed some of his best letters on philosophy. We extract the following particulars from a letter written by this estimable person to Dr. Simmons, and originally published by him in his life of Dr. Hunter.

"Mr. Hewson was born at Hexham, in Northumberland, on the 14th Nov. O. S., 1739. He received the rudiments of his education at the grammar-school in that town under the Rev. Mr. Brown. His father was a surgeon and apothecary in the place, and much respected in that neighbourhood. With him Mr. Hewson acquired his first medical knowledge. Being ambitious to increase that knowledge, he placed himself first under an eminent surgeon in Newcastle, Mr. Lambert, and afterwards resided some time at London, Edinburgh, and Paris. His subsequent acquirements are sufficient to prove that he visited those places with a true love of science and desire of attaining eminence in his profession.

"A better son and husband, or a fonder father than Mr. Hewson never existed. He was honoured with the friendship of many respectable persons now living, and the late Sir John Pringle showed him singular marks of regard.

"Mr. Hewson's manners were gentle and engaging; his ambition was free from ostentation, his prudence was without meanness, and he was more covetous of fame than of fortune." P. xiv–xv.

The details of the professional career sketched in this letter are soon related. In 1759, Hewson came to London, lodged with John Hunter, and attended the Anatomical Lectures of Dr. William Hunter, at a house in Covent Garden.

"Hewson's diligence and skill soon recommended him to the favorable notice of the brothers; and when Mr. Hunter went abroad with the army, early in 1761, he left to Mr. Hewson the charge of instructing the other pupils in the dissecting-room, by which means, as Mrs. Hewson remarks, he gained money at an age when most students in surgery are only spending it.

"He entered himself a pupil at Guy's and St. Thomas's Hospitals: and attended Dr. Colin Mackenzie on midwifery, and Dr. Hugh Smith on physic. In gratitude to the liberal confidence of his father, Hewson was justly careful of his money, and this prudence continued through life, though not so as to check the growth of generosity; for no man ever exercised his profession with less avidity of gain; he disdained every species of meanness, and possessed a judicious liberality which elevated his character. His father had the happiness of living to reap the fruit of this care." P. xv.

Hewson subsequently (in 1762) studied at Edinburgh, and on his return entered into partnership with Dr. Hunter. In the Summer of 1765, he visited France; on March 8, 1770, was elected a Fellow of the Royal Society; and in the November of the same year, he received the Copley Gold Medal "for his papers on the lymphatic system in birds, amphibious animals and fishes." In 1769, Dr. Hunter finished his building in Windmill-street, where Hewson had a small apartment allotted to him. Mr, Gulliver has very appropriately alluded to this celebrated place in the following words:—

"The Windmill-street School no longer exists; but it will be preserved from oblivion by the names of the eminent men who lectured there. Among these the future historian of anatomy and physiology in England will have to commemorate William Hunter, Hewson, Cruikshank, Sheldon, Baillie, Brodie, Charles Bell." P. xvi.

We now approach the close of these brilliant prospects, which are thus depicted by a distinguished contemporary. "In viewing the situation of our associate at this period," says Dr. Lettsom, "the most gratifying prospects presented themselves, where genius and industry were rewarded with success, and domestic amities with felicity. The theatre in which he delivered his lectures and expounded his doctrines was crowded with men of science, as well as with pupils, to listen to a youth grown sage by experimental researches."—(P. xviii.) But this happiness was soon to end. He was seized with a fever, occasioned by a wound received in dissection, which proved fatal on May-day 1774, after a short illness, in the thirty-fifth year of his age.

He was buried at St. Martin's-in-the-Fields; but the only record of this admirable physiologist, whose life thus fell a sacrifice to his unceasing zeal for the advancement of science, is a meagre entry in the parish register. Surely some more honorable memorial should hand down to posterity the name and sterling merits of one of the brightest ornaments our profession has ever produced. If the proposition were once judiciously made, we have no doubt funds would readily be found to erect a tablet to the memory of a man equally esteemed in private and in public life.

Subsequently to the death of her husband, Mrs. Hewson continued to enjoy the esteem of Dr. Franklin, the friend of her youth, of which we have a pleasing proof in the following letter, addressed to her from Passy, and bearing date, January 27th, 1783.

"In looking forward, twenty-five years seem a long period, but in looking back, how short! Could you imagine, that it is now full a quarter of a century since we were first acquainted? It was in 1757. During the greatest part of the time I lived in the house with my dear deceased friend, your mother; of course you and I conversed with each other much and often. It is to all our honours, that in all that time we never had among us the smallest misunderstanding. Our friendship has been all clear sunshine, without the least cloud in its hemisphere. Let me conclude by saying to you, what I have had too frequent occasions to say to my other remaining old friends. The fewer we become, the more let us love one another."—*Introd.* p. xx.

After Mrs. Hewson lost her mother, and in compliance with the frequently expressed wish of Franklin that she should become his neighbour in America, she went with her children to Philadelphia, and died in 1795, leaving two sons and a daughter. The second and surviving son, Dr. Thomas Tickell Hewson, is, according to inquiries lately made by Mr. Gulliver, still alive, and is the President of the College of Physicians at Philadelphia.

Having given this brief biographical notice, which we are assured our readers will agree with us was a proper tribute to the memory of such a man as Hewson, we proceed to the consideration of his scientific labours, the principal of which relate, as it is well known, to the blood. In order to do justice to these researches, it will be necessary to enter somewhat fully into the properties of the circulating fluid: we trust, however, that this detail may not be devoid of interest in another point of view, as the volume before us is enriched by copious notes, in which the judicious editor has brought together a large amount of important matter, collected from various sources, his own inquiries forming a very valuable portion of these additions.

The very interesting and comprehensive introduction of Mr. Gulliver, in which he traces the successive progress of discovery in relation to the blood, shows how much we are indebted to Hewson for an exact knowledge of many of the fundamental qualities of the circulating fluid; and on how many points he had anticipated the results of modern discovery. It is indeed true in this, as in so many other instances, that several facts of great importance had been discovered by earlier observers; but then owing, partly, to the false hypotheses with which they were encumbered; and, partly to the neglect and errors of subsequent writers, the whole history of the constitution of the blood was involved in great confusion when our author commenced his "Experimental Inquiries." Upon this point Mr. Gulliver justly remarks that—

"In considering the labours of Hewson in connexion with the facts observed and the errors held by his predecessors and contemporaries, it must be recollected that the speculations connected with Leeuwenhoek's microscopical researches for many years supplanted accurate experimental inquiries into the properties of the blood; so that the fibrin was either forgotten or confounded with the serum, and a fanciful importance was given to the red corpuscles. When the errors consequent on this state of things began to wane, the blood sunk into neglect. Accordingly, the just observations of Malpighi, Lower, and Borelli, were lost for the greater part of a century, while the coagulation of the blood was ascribed to a mere cohesion or running together of the red corpuscles, and the formation of fibrinous clots to a change in the serum. These opinions were held in Britain by the best writers to the year 1760, and on the Continent by the most distinguished physiologists, as Haller and Marherr, up to or even beyond 1771, the date of the first edition of Hewson's 'Inquiry into the Properties of the Blood.' But there were exceptions. The knowledge of Petit, Quesnay, Senac, and Gaubius, was unquestionably in advance of that current in their day; yet they added but little to the facts of the older observers, so vaguely discussed by Haller, so ably used in the masterly memoir of Petit; and some of the opinions set forth by Quesnay and Senac, partook of that general crudity which proved how necessary it was that the properties of the blood should be studied anew by the experimental method."—*Introd.* p. xl.

It would be unjust towards a neglected but ingenious observer, Dr. Richard Davies, if we did not state that, in the opinion both of Dr. Davy and of Mr. Gulliver, to this writer, who preceded Hewson by about twenty years, belongs the merit of clearly apprehending the true method of studying the blood, namely, by experimental research. His "Essay," considered as a demonstration of the distinctive characters of the three proximate principles of the blood, is also, we are informed by the same authority, "admirably decisive."

Although the independent existence of these three constituents, the fibrin, the red corpuscles, and the serum, and their relation to the process of coagulation, are for the most part well known, yet, in order to guard against the errors which are even in the present day, from time to time, springing up; and especially for the purpose of adducing a few of those simple but convincing experiments which are a model in this field of research, we quote the following from the first part of the "Experimental Inquiries." Alluding to the inflammatory crust, and to the opinion entertained by some authorities that it is formed by the serum, Mr. Hewson remarks:

"But that it is formed by the coagulable lymph alone, after the red particles have subsided, appears from the following experiments.

"*Experiment* 11.—In the month of June, when the thermometer in the shade stood at 67°, I bled a man who had laboured under a phthisis pulmonalis for some months, and at that time complained of a pain in his side. The blood, though it came out in a small stream, yet flowed with such velocity, that it soon filled the basin. After tying up his arm I attended to the blood, and observed that the surface became transparent, and that the transparency gradually went deeper and deeper, the blood being still fluid. I likewise observed that the coagulation first began on the surface, where it was in contact with the air, and formed a thin pellicle; this I removed, and saw that it was soon succeeded by a second. I then took up a part of the clear liquor with a wet teaspoon, and put it into a phial with an equal quantity of water; a second portion I kept in the teaspoon; and I found afterwards that they both jellied or coagulated, as did also the surface of the crassamentum, making a thick crust. On pressing with my finger that portion which was in the teaspoon, I found it contained a little serum.

"From this experiment it is evident, that the substance which formed the size was fluid after it was taken from the vein, and coagulated when exposed to the air; and as this is a property of the coagulable lymph alone, and not of the serum, there can be no doubt that the size was formed of the lymph." P. 32.

In order further to demonstrate the same fact, Hewson performed many interesting experiments, in which, having observed that neutral salts have the power of preventing the process of coagulation, he availed himself of this circumstance to obtain the fibrin or coagulable lymph, as he terms it, separately from the red corpuscles.

"In these mixtures of the blood with neutral salts, the red particles readily subside, (especially if human blood be used), and the surface of the mixture becomes clear and colourless; and being poured off from the red part, it is found to contain the coagulable lymph, which can be separated by the addition of water." P. 12.

After all these, and a great number of other exact experiments, it is strange that any doubt should subsequently have arisen as to which was the coagulable matter of the blood. We need, however, scarcely remind our readers, that Sir E. Home and Mr. Bauer, reviving what Mr. Gulliver calls the old and discarded hypothesis of Sydenham, contended that the fibrin, instead of being what Hewson had so distinctly proved it to be, an independent constituent, was formed in the act of coagulation by the colourless matter of the red corpuscles. This error, which received little or no sanction from English physiologists, became so prevalent on the Continent, that Professor Müller was deemed to have rendered good service for proving, partly, by the very ingenious experiment of mechanically separating by filtration the red corpuscles from the fibrin in the blood of the frog; and, partly, by repeating some of Hewson's experiments with the neutral salts, that the coagulating material is altogether distinct from the colouring substance. Another theory has also been lately advocated, to the effect that both the fibrin and the albumen of the serum are contained within the pale or colourless corpuscles of the blood; a position which we believe to be utterly untenable. Whether it be true, as some physiologists contend, that these corpuscles elaborate the plastic element or fibrin; or that this change is effected by the red corpuscles; or that the fibrin is formed in some other and at present unknown way, which, considering the chemical relations of that substance and of albumen, may be regarded as the most probable explanation; however this may be, there can, we think, be no reasonable ground for doubting that, once formed, the fibrin exists as an independent principle, composing, in conjunction with the serum, the fluid portion of the circulating blood, or liquor sanguinis.

A point on which Hewson made many valuable observations, relates to the production of the buffy coat; and as this is a subject of practical importance, and one, moreover, that has received much elucidation from modern research, we are desirous of stating the results that have been obtained. It is a striking illustration of the accuracy which characterises Hewson's investigations, that he detected one of the most essential changes of buffy blood, namely, its attenuation, and this in opposition to the prevailing opinion.

"How contrary," he says, "to the conclusion, which these experiments lead us to, are the opinions of some medical writers on this subject! How frequently do we find it said, that the blood is thicker in inflammatory disorders, where that size occurs; and that a large orifice is necessary to let out the vitiated blood! That a large orifice is preferable to a small one in many cases, where such blood is found, I believe to be true; but that its advantages are owing to its letting out the thickened blood, seems improbable from what we have seen in the experiments above related: they are perhaps nearer the truth, who attribute it to the suddenness of the evacuation." P. 40.

He further ascertained the fact, that the red corpuscles sink more rapidly in the liquor sanguinis of buffy blood than in the serum alone; and, putting these circumstances together, it is not a matter of surprize, that he attributed the formation of the sizy coat to the attenuation of the liquor sanguinis, and to the more ready subsidence of the red corpuscles consequent thereon towards the lower part of the crassamentum.

The application of the microscope has thrown a clearer light upon the phenomenon in question; and has shown that the peculiar attraction which, under certain circumstances, the red corpuscles have for each other, and in virtue of which they form the well-known piles or rouleaux, plays the most essential part in the formation of the buffy coat. This piling of the red discs, which is one of the most remarkable, and at the same time beautiful, appearances seen on examining a drop of blood,* was evidently known to Hewson; for he remarks, in arguing for the flat form of the corpuscles, "I have seen them with their sides parallel, like a number of coins laid one against another."—*L. c.*, p. 228.

Dr. Hodgkin and Mr. Lister were the first in the revival of microscopical anatomy, distinctly to describe the disposition just noticed, which takes place in healthy blood during coagulation. But what more particularly concerns the present question is that, in buffy blood, the red corpuscles evince an increased tendency to aggregation; a fact well known to Hunter, who, in speaking of the blood in inflammation, says, "the red globules become less uniformly diffused, and their attraction to one another becomes

---

* The piling of the corpuscles affords to the inexperienced microscopist, the best idea as to how the red colour of the blood is produced; for it is seen that, when the piles are formed, the so-called red corpuscles, which are, as it is familiarly known, of a pale yellow examined singly, begin from their aggregation to reflect the red ray, and to assume a pinkish tint; and that where two rouleaus lie across each other, the tint becomes deeper; till at last, where several piles are heaped together, the ordinary red colour of the blood is produced.

stronger;" so that the blood, when out of the vessels, soon becomes cloudy or muddy, "and when spread over any surface, it appears mottled, the red blood attracting itself and forming spots of red." In this passage, as Mr. Gulliver justly remarks, Mr. Hunter has anticipated some recent observations; and if he had employed the microscope, which instrument, however, there is reason to suppose, he did not use, he would have seen, as Dr. H. Nasse was the first to show, that in buffy blood the red corpuscles more readily run together into piles, and, consequently, separate from the fibrin more completely; circumstances which are apparently favoured by the diminution of the corpuscles, and the increase of the fibrin occurring in inflammation. It is then this augmented attraction of the blood-discs for each other that ought to be regarded as the essential condition on which the sizy coat depends; though there is no doubt, as Mr. W. Jones has explained, that the massing together just noticed, increases the specific gravity of the corpuscles, considered in the aggregate, and thus allows of gravitation coming into play as a secondary cause. This is well put by Mr. Gulliver, who remarks, "in buffy blood, they (the corpuscles) are still more closely connected, almost as if fused together, and the piles run into little clumps often visible to the naked eye. Now we know that coarse particles sink more rapidly in a liquid than fine ones, and the clumps of corpuscles in buffy blood represent much coarser particles than their composing single piles or separate corpuscles. The more they run together, therefore, the faster they will fall in the liquor sanguinis, so as to produce the buffy coat. Accordingly, it was found in my experiments that, during the formation of the buffy coat, the corpuscles will at first take about two and a half minutes to sink an eighth of an inch in the liquor sanguinis, and fall five or six times faster in the next two or three minutes; and that this remarkable acceleration in the sinking of the corpuscles is connected with their increasing aggregation, appeared from the fact that their rapidity of sinking was increased by increasing their aggregation, though the means used did not attenuate the blood; and prevented or even reversed by destroying the aggregation, though by means which made the blood much thinner and lighter."—P. 41, *Note* xxiii.

In connexion with this subject, it may not be superfluous to notice that the buffy coat, although so frequently an accompaniment of inflammation, is not to be regarded as an invariable sign of that state; thus it occurs regularly in the blood of the horse, and also, as it is well known, in the advanced stage of pregnancy. In these instances there is an excess of fibrin, and it is probable that this circumstance may, as the majority of writers suppose, favour the formation of the buffy coat. It may, also, be as well to state that the peculiar piling together of the red corpuscles is not confined to the coagulating process, having been observed by several microscopists to supervene in the small vessels of inflamed parts, producing an appearance in the stagnant blood, as if the corpuscles were fused together; and unless the circulation is restored to its normal state, doubtless influencing, in an important manner, the subsequent phenomena.

There is no part of these works which may be perused with more advantage than that which treats of the influence of faintness on the process of coagulation, and of the principles that should be observed in the treatment of hæmorrhage. It seems that Hewson was led to investigate this subject by an observation of Dr. Hunter to the effect, that the faintness which comes on after hæmorrhage, instead of alarming the byestanders, and inducing them to support the patient by stimuli and cordials, " should be looked upon as salutary, as it seems to be the method Nature takes to give the blood time to coagulate"

Suspecting that the disposition to coagulate was increased in those cases where the vital powers were weakened, the author performed the following conclusive experiment :—" Believing it would be sufficient for this purpose to attend to the properties of the blood, as it flows at different times from an animal that is bleeding to death, I therefore went to the markets, and attended the killing of sheep ; and having received the blood into cups, I found my notion verified. For I observed, that the blood which came from the vessels immediately on withdrawing the knife was about two minutes in beginning to coagulate ; and that the blood taken later, or as the animal became weaker, coagulated in less and less time ; till at last, when the animal became very weak, the blood, though quite fluid as it came from the vessels, yet had hardly been received into the cup before it congealed. I have also repeated the experiment, by receiving blood into different cups at different times, whilst the animal was bleeding to death ; and though the time taken up in killing the animal was not commonly more than two minutes, yet I observed, on comparing the cups, that the blood which issued last coagulated first." *L. c.*, p. 46.

The truth of this conclusion, shaken for a time by the experiments of the late Mr. Hey, was fully established by the valuable researches of Mr. Thackrah ; and must ever be regarded, in respect to the means of suppressing hæmorrhage, as one of the most fundamental principles connected with the blood. Although so much has been since written on this subject, the admirable observations of Hewson leave little to be desired.

" As hæmorrhages," he says, " seem to be stopped, partly by a contraction of the bleeding orifices, and partly by the coagulation of the blood, and as the disposition of the blood to coagulate is increased by weakening the body, and likewise the contraction of the bleeding orifices is promoted by the same means, it is therefore evident that the medicines to be used should be such as cool the body, and lessen the force of the circulation ; and experience teaches us, that such are the most efficacious. It likewise shows that all agitation of mind and all bodily motion should as much as possible be prevented ; because they increase the force of the circulation, and are thence unfavourable to the stopping of the hæmorrhage. But that languor and faintness being favourable to the coagulation of the blood, and to the contraction of the bleeding orifices, should not be counteracted by stimulating medicines, but on the contrary, should be encouraged. And as evacuations weaken the body more when they are sudden, we see a reason why blood-letting should be advisable in hæmorrhages, and why a large orifice should be preferable to a small one, when we want to produce that languor or faintness, or that weak action of the vessels, so useful for the stopping of the hæmorrhage." P. 77.

There is an interesting point connected with the coagulation of the blood ; namely, that the process is retarded or entirely prevented, so long as the blood is in contact with the living tissues ; not merely with the blood-vessels, as where Hewson tied up the jugular vein of a dog and found but a very slight coagulum at the end of two hours and a quarter ; but even occasionally in cases of extravasation. Thus, Mr. Gulliver mentions the case of a soldier, who had received a contusion in his loins, and in which five ounces of blood, evidently effused at the time of the accident, was let out twenty-eight days afterwards and found to be as liquid as if just drawn from a vein, though it coagulated in a cup in less than thirty minutes. The experience of most observers and practitioners must have furnished similar instances.

The authority of Mr. Hunter is so influential in all points relating to the blood, that it is particularly necessary to correct any errors into which he may have fallen; we therefore would call the attention of our readers to the following observations of Mr. Gulliver, relative to a point of some physiological interest.

"The causes usually given, on the authority of Mr. Hunter, as altogether destroying the coagulable property of blood and the contractility of muscle, are some of them so doubtful that they all require to be examined anew. Thus, in a man killed by lightning, Dr. Davy observed some soft coagulum in the heart, and that the fingers were rigid, although the examination was not made until the body was rather advanced in putrefaction. Sir Charles Scudamore invariably found the blood coagulated as usual in animals killed by electricity. Sir B. Brodie found that the irritability of the muscular fibre was not destroyed in a guinea-pig which had been instantly killed by an electric shock.

"Dr. Andrew Smith commonly saw coaglated blood in the hearts of antelopes run down by dogs. In a hunted hare Dr. Davy saw, he informs me, some coagulated blood. So did I in one that had been run for thirty-five minutes and then killed by the Windsor harriers. * * * Finally, as to a blow on the stomach: In a cat killed by a kick, which ruptured the stomach and liver, I found coagulated blood in the heart, and the limbs rigid, seventeen hours after death." P. 21.

We proceed to notice that constituent of the blood, which, since the invention of the microscope, has ever been a favourite branch of research, and to which a large part of Hewson's writings is devoted; we allude to the coloring matter, composed of the red corpuscles. Discovered originally by Malpighi, and affirmed erroneously by Leeuwenhoek, to be globular, the red corpuscles were first demonstrated to be flattened discs by Hewson. The method by which he ascertained this fact evinces his usual tact and discrimination: being convinced that former observers had failed to detect the true figure of these bodies in consequence of their being so much crowded together, that the blood, when viewed through the microscope, appears a confused mass, it occurred to him to dilute it with serum, in which the discs remain unaltered. In making this observation, it is necessary, as Mr. Gulliver points out, to examine the corpuscles in their own serum; for so subtle are the influences operating on them, that it may be difficult to distinguish correctly their outline in the serum of another animal, which is apt to cause them to aggregate into clumps, as when the serum of the horse is added to the blood of other mammals, and, in consequence, as it would seem, of some difference as to the amount of saline matter.

It is well known that whilst in mammalia and man the red corpuscles are circular and biconcave, in the oviparous vertebrata, they are elliptical and bi-convex; there are, however, some remarkable exceptions to this general principle, for in the camelidæ among mammals the corpuscles are *oval*, whilst in cyclostomatous fishes and amphioxus, the discs are circular; but in both of these instances there is no deviation as to structure, the oval corpuscles of the camel, for example, agreeing with those of other mammalia in having no nucleus (Gulliver), whilst those of the lamprey have that characteristic of the oviparous vertebrata (Wharton Jones).

The question relating to the organization of the red discs, for that they are organized bodies has long been known in a general way, and has been specially demonstrated in late years, requires that we should refer to the first formation or development of the corpuscles; a subject minutely examined by Hewson, and which has subsequently received much attention, especially in the elaborate memoirs of Mr. Wharton Jones, lately published in the Philosophical Transactions, entitled "The Blood-corpuscle in its different phases of Development in the Animal Series." The fully-formed red corpuscle consists, according to the best observers, of a colourless envelope or vesicle, and of coloured contents; in the oviparous vertebrata, but not in mammals or man, there is a nucleus, which is insoluble in water or acetic acid. Hewson, it is well known, confidently contended for the existence of a nucleus in mammalia; this opinion is strongly expressed in the following passage:—"From the greater thickness of the vesicles in the human subject, and from their being less transparent when made spherical by the addition of water, and likewise from their being so much smaller than those of fish or frogs, it is more difficult to get a sight of the middle particle, rolling from side to side in the vesicle, which is become round; but with a strong light, and a deep magnifier, I have distinctly seen it in the human subject, as well as in the frog, toad, and skate."—(P. 224). He further affirmed that the nucleus is white, and the vesicle red.

Although, as we have stated above, the nucleus is apparently wanting in mammalia, yet it was necessary to state Hewson's opinion, because it is intimately connected with the celebrated theory he so strenuously advocated respecting the formation of the red particle. This theory briefly stated is, that the red corpuscles are formed by the lymphatic system and its appendages, consisting, according to the author, of "the lymphatic vessels, lymphatic glands, the thymus, and the spleen:" of these organs, the lymphatic glands and the thymus, so long as it persists, form the white nuclei or "central particles," as Hewson terms them, whilst the lymphatic vessels and the spleen form the red vesicles.* No one who has not carefully considered the important body of evidence adduced by Hewson in support of his doctrine, or who is unacquainted with the minute anatomy of the present day, is qualified to pronounce an opinion upon its value; and no one, we may confidently affirm, who has thus studied his profound researches, whatever may be the conclusion at which he may arrive as to the particular fact of the formation of the red corpuscle, can rise from their perusal without feelings of admiration for their author, and of surprize that he should on so many points have closely approached some of the deepest truths of structural anatomy. Our limits will, however, only allow us to make a few extracts. Hewson proved that the lymphatic

* Hewson erroneously supposed that the seat of colour was in the envelope of the red corpuscles, and this mistake has been repeated by some writers of the present day.

glands are secreting organs, and, in the following paragraph, has indicated their true structure, lately more precisely demonstrated by one of the most successful observers of the present day, Mr. Goodsir.

"The arteries and veins are principally spread on the coats of the lymphatic vessels, so that we here find the requisites to form a lymphatic gland; for as we prove that many of the lymphatic glands in the human body are no more than a congeries of arteries, veins, nerves, and lymphatic vessels convoluted, it is probable that all lymphatic glands may be formed in the same manner; so, perhaps, it may be the same thing in nature, or the same purposes of the animal economy may be equally well answered, whether the parts composing a gland (viz. arteries, veins, nerves, and lymphatic vessels) be circumscribed in a proper membrane, or spread over a large surface. This, perhaps, will be more fully proved by some experiments and observations which I shall hereafter publish on the minute structure of glands." *L. c.* p. 251.

It is Mr. Falconar, the editor of Hewson's posthumous works, who is here speaking for his friend; unhappily he did not live to fulfil this promise, having died very shortly afterwards.

The peculiar secretion of the lymphatic glands, consisting of the now well-known lymph-corpuscles, is thus described:

"On cutting into a fresh lymphatic gland we find it contains a thickish, white, milky fluid. Then if we carefully wipe or wash this fluid from any part of the cut surface, and examine it attentively in the microscope, we observe an almost infinite number of small cells, not such as have been before described, or that have been supposed to exist in the lymphatic glands, but others too small to become visible to the naked eye." P. 251.

They are further on spoken of as "small, white, solid particles;" and the lymphatic vessels are said to be analogous to the excretory ducts of other glands, the proof being that, if a ligature be made on the lymphatic vessel coming from a gland, a fluid is found of the same kind as that contained in the gland itself. But other and more direct indications are adduced: thus, it is repeatedly asserted that red corpuscles were seen in the fluid taken from the lymphatic vessels after they had passed through the glands; that when a ligature had been applied around the vessels of the spleen, in an ox first killed, "the lymphatic vessels soon became turgid, and were distinctly seen filled with a red fluid," and on diluting some of this fluid with a weak solution of Glauber's salts, exactly the same appearances were exhibited as those seen on similarly examining the blood. (P. 272.) An important fact corroborative of Hewson's theory, may be here adduced; it is that the chyle unquestionably acquires its peculiar corpuscles as it traverses the mesenteric absorbent vessels and especially the glands; so that either these lacteal vessels and glands must be the formative organs of the chyle-corpuscles, which are on the average somewhat smaller than the red discs (their diameter being about $\frac{1}{4600}$ of an inch); or these corpuscles must be the result of certain molecular changes accomplished by the chyle itself.

Although the experiments which are affirmed to prove the existence of red corpuscles in the lymphatics, are not free from objection; and may in part be explained by the circumstance first noticed by Mr. Lane, that in opening the absorbents, some small blood-vessels are wounded and so allow their red discs to become mixed with the lymph; still the fact contended for by Hewson, has been confirmed by too many accurate observers to be any longer a matter of doubt. The principal evidence hitherto obtained on this point is thus summed up in Mr. Gulliver's notes.

"Red corpuscles are certainly sometimes found in the lymphatic vessels, and generally in those of the spleen of the horse and ox; but it would appear that the reddish colour of the splenic lymph is not constant. Mr. Lane found the ruddy colour of the horse's chyle due to the presence of red corpuscles; and he and Mr. Ancell observed imperfect blood-corpuscles, and attributed the rose-colour of the lymph to them, in the large lymphatic vessels. The thoracic duct of the horse often appears as a coloured tube from the number of these corpuscles in the chyle, which, as described in the Appendix to the English edition of Gerber's 'Anatomy,' p. 93, I have generally found to be smaller, more irregular and less perfect in shape, than the red corpuscles in the blood; and the same observation is applicable to the red corpuscles in the splenic lymph of this animal. Dr. Simon's observations on red corpuscles in the thoracic duct of the rabbit and horse, are to the same effect. Schultz and Gurlt also noticed the chyle of a reddish colour from the presence of blood-corpuscles, of which they suppose, with Simon, the formation to begin in the chyle. The transition of the corpuscle of the chyle or lymph into the red corpuscle of the blood seems now to be commonly admitted in Germany. Dr. Davy informs me that he found a small portion of red crassamentum in the thoracic duct of a man who died suddenly of apoplexy." *Note* CXLI., p. 276.

Hewson's theory, as he himself readily perceived, is open to the objection that the spleen may be removed in a living animal without preventing the formation of the red particles. His answer is, that this organ is not the only one provided for the formation of the vesicular portion, the lymphatic vessels being endowed with the same power;—"Nature has given the spleen as an auxiliary to the lymphatic system, in order to the more commodiously, expeditiously, and completely forming the red part of the blood." In the same way with respect to the thymus, which exists only in the early period of life, although its agency is at that active time required "for the purpose of forming more of the central particles of the blood than could have been made by the lymphatic glands alone," yet subsequently when the demand is less, the thymus can be dispensed with, the absorbent glands being then competent to supply the central nuclei. Mr. Gulliver justly observes, in reference to this latter subject, that Hewson's observation to the effect "that the lymphatic vessels of the thymus do carry a fluid, however it may get into them, like that of the thymus, and pervaded by the same globules," has never been refuted; and he quotes the authority of Sir A. Cooper, that the lymphatic vessels are the absorbent ducts of the gland, and the carriers of its fluid into the veins of the lower part of the neck.

It will not be superfluous to remark in this place, that this, the most profound and important of all Hewson's researches, could not be properly appreciated at the time when it was announced: it must be viewed in connexion with the cell-theory, with which the formation of the "central particles" (nuclei) and of the enveloping "vesicles" (cells) has a clear and distinct relation.

The minute and elaborate microscopic observations of Mr. Wharton Jones, which immediately bear upon the question we are considering, are worthy of the most careful attention. The object of these memoirs is to prove that the red corpuscle passes through several successive stages in its development before it is fully formed. In its first and simplest form, the future red disc exists as a perfectly colourless corpuscle termed "granule blood-cell," consisting of a vesicle with granular contents, which latter may be either of a coarse or fine character; these "granule-cells" have a diameter in the embryo ox varying from about $\frac{1}{1460}$ to $\frac{1}{1825}$ of an inch; and in the adult human body, when distended with water, their diameter is about $\frac{1}{2400}$ of an inch. The contained granules are extremely minute, varying in diameter according to the degree of development from $\frac{1}{15000}$ to $\frac{1}{25000}$ of an inch, and presenting, as in other similar instances, active molecular motion: a nucleus may be detected by treating the granule-cell with acetic acid. In the second and more advanced stage, distinct nucleated cells appear, which are at first colourless, but subsequently attain the yellow tint proper to the fully-formed corpuscles. Thus, the author recognizes two "phases" or grades of development in oviparous vertebrated animals, namely, fishes, reptiles, and birds, each phase having two stages; the process of formation may be thus expressed:

| | |
|---|---|
| First phase, granule-cell | 1st stage, coarsely granular.<br>2nd stage, finely granular. |
| Second phase, nucleated-cell | 1st stage, uncoloured.<br>2nd stage, coloured. |

The fully-formed blood of the mammifera and man, contain, like the preceding classes, granule-cells, both coarsely and finely granulated, and nucleated blood-cells in the uncoloured stage also exist; it is to these three forms of cells that the somewhat vague name of "lymph" or "colourless" corpuscle has been applied. In mammiferous animals, especially in the horse and elephant, nucleated blood-cells in the coloured stage, have been found; but such corpuscles have not been seen in the fully-formed blood of man unacted on by re-agents. One of the most novel views contained in these memoirs relates, however, to the existence in mammalia and man of a third and higher phase of development, not possessed by the oviparous vertebrata—that of the "*cellæform nucleus,*" constituting the red corpuscle of other anatomists, the nature of which will be understood by the following explanation.

The author conceives that physiologists, misled by the similarity of colour, have taken for granted, that the red corpuscle of mammalia is the exact analogue of the red corpuscle of the oviparous vertebrata; whilst, as he contends, it really represents only the *cellæform nucleus* of that body.

"The view of the nature of the 'red corpuscle' of the fully-formed blood of Man and the Mammifera to which I refer is this: the 'red corpuscle' of the fully-formed blood of Man and the Mammifera is the cellæform nucleus of the nucleated cell set free by the bursting of this cell itself, and become filled and red by the secretion of globuline and colouring matter into its interior."—*Phil. Trans.* p. 75.

This explanation of the nature of the red corpuscles in mammals is thought by Mr. W. Jones to clear up the long-disputed question as to the existence or non-existence of a nucleus in that class of animals. He says:

"Physiologists have accordingly supposed that it (namely, the red corpuscle of mammalia) should contain a nucleus; but though unsuccessful in the attempt to demonstrate one, they have not altogether ceased to believe in the existence of an exact analogy between it and the 'red corpuscle' or coloured nucleated blood-cell of the oviparous Vertebrata; they have rather had recourse to conjecture to account for the absence of a nucleus."—*L. c.*, p. 74.

In order to form an opinion of the value of this theory, it is necessary carefully to consider the constitution of the blood in the embryo of mammiferous animals and man. It is well known to physiologists that the blood, which at first circulates in the embryo, is colourless, and, which is a most interesting fact, that the blood-corpuscle has at that epoch a nucleus; moreover, there is an abundance of free globules, which are said by some writers to resemble the nuclei of the nucleated corpuscles just noticed. Among these peculiarities of the embryonic blood, the most important is the existence of a nucleated cell; and the questions immediately suggest themselves, as such cells certainly do not exist in the fully-formed blood of the adult, what is their signification, and what becomes of them? These queries have been answered in one sense by that excellent observer, Mr. Gulliver, who considers, as regards the first point, that the nucleated blood-cell of the mammiferous embryo, is the exact analogue of the red corpuscle or coloured nucleated blood-cell of the oviparous vertebrata; whilst, with respect to the second question, he affirms that "in mammals the nucleus of the red blood-corpuscle soon disappears," and in this way the above-noticed nucleated cell is transformed into the non-nucleated red corpuscle of the adult mammals. From what has already been stated, it appears that Mr. W. Jones offers a very different explanation of this admittedly difficult point of minute anatomy; this writer contending that the nucleated cells burst, the nuclei alone remaining and becoming the red corpuscles of mammalia. In reference to Mr. Gulliver's view of the subject, Mr. Jones observes, with much justice, that "this apology for the absence of a nucleus in the 'red corpuscle' of the fully-formed blood of Man and the Mammifera, would have had weight if the 'red corpuscle' had been an object persistent throughout life, like a limb or an eye, but as it is an object constantly disappearing and being regenerated, we should expect, if it were really a nucleated cell originally, to meet with it in a stage when it does contain a nucleus." *L. c.*, p. 74.

We cannot quote the reasons adduced by the author in support of his opinions; but it is due to this patient and successful investigator to observe that the facts brought forward have much force, and must form an important element in this question till it is finally settled. Whether his peculiar views of the constitution of the red particles in the fully-formed blood of mammalia be established or not, must very much depend upon the nature of that essential part of the nucleated cell, the nucleus. That this possesses essentially a limiting wall or vesicle, is generally agreed; and thus we have one of the most indispensable conditions, the existence, namely, of an organic membrane, for the production of those endosmotic phenomena attributed by Mr. W. Jones to the so-called cellæform nucleus. In the present aspect of this question, with every inclination to receive with candour the researches of this eminent physiologist, and fully admitting the force of his observations upon the correspondence in size, between the nucleus of the nucleated blood-disc and the red corpuscles of the several mammalia whose blood was examined, we conceive that further researches are required to arrive at a satisfactory conclusion.

We regret that our limits will only allow us to make one or two additional extracts from these important " memoirs." The examination of the avertebrate animals has established the important fact, that, contrary to the opinion of those physiologists who conceive that in these classes the nutritive fluid corresponds to the chyle or lymph of the vertebrata, the blood contains true blood-corpuscles, though not in the same degree of development as in the vertebrate division of the animal kingdom. In the erab and in the invertebrata downwards, so far as the investigation has been carried, granule-cells in both the coarsely and finely granular stages, and also nucleated cells in the uncoloured stage, exist; even a slight appearance of colour is detected in the nucleated cells of the crab and lobster. An interesting fact was ascertained by Professor Graham on analysing some of the corpuscles of the crab; namely, that, although colourless, " they contained a sensible quantity of iron, perhaps as much as red corpuscles;" thus corroborating the conviction now prevalent among physiologists, that the red colour does not depend on the presence of iron, and also supporting the theory of Liebig respecting the part played by the iron of the blood-corpuscles in the development of animal heat. The general conclusions of the author respecting the blood of the invertebrata, when compared with the circulating fluid of the oviparous vertebrata and the mammifera, are thus expressed.

" In the oviparous Vertebrata, from the Skate upwards, it has been seen that the blood-corpuscle in its different phases of development is essentially similar to that of the Skate. In the Invertebrata, from the Crab down as far as we have gone, it has also been seen that the blood-corpuscle in its different phases of development is essentially similar to that of the Crab. The only difference therefore in essential respects between the blood-corpuscle of the oviparous Vertebrata generally and that of the Invertebrata, is the same as that between the blood-corpuscle of the Skate and Crab, viz. that in the phase of nucleated cell, the latter does not attain to a decidedly coloured stage. In the phase of granule-cell, the blood-corpuscle of the Mammifera and that of Invertebrata resemble each other in essential points of structure. In the phase of nucleated cell they also agree in the absence of a decided coloured stage: in this respect differing from the blood-corpuscle of the oviparous Vertebrata generally and of the early mammiferous embryo. But here the resemblance between the blood-corpuscle of the Mammifera and that of the Invertebrata ceases. In common with the blood-corpuscle of the oviparous Vertebrata, that of the Invertebrata differs from the blood-corpuscle of the Mammifera in not attaining to a decided third phase." *L. c.*, p. 104.

The account given by Mr. Jones of the corpuscles existing in the lymphatic vessels of man and the mammifera, is of peculiar interest, when taken in connexion with Hewson's description of the genesis of the red corpuscles; it may be proper to premise that the fluid for examination was taken from the thoracic duct.

" The corpuscles were,—1st, Granule-cells in both coarsely and finely granular stages, altogether similar to those of the blood. 2nd. Besides many cells in transition from granule to nucleated phase, nucleated cells in both uncoloured and coloured stages, quite the same as those of the blood. The nucleated cells were fully distended independent of the addition of water, and were comparatively the most numerous of the corpuscles in the lymph; those in the coloured stage being more so than those in the uncoloured stage. Slight as the coloration of the coloured stage of the nucleated cell was generally, some few cells presented it perhaps in a more marked manner than is usually presented by the same cells as they occur in the fully-formed blood; in this respect approaching to the red nucleated cell of the blood of the early mammiferous embryo. Some even were of an oval shape. 3rd. A considerable number of free cellæform nuclei, both uncoloured and in different degrees of progressive coloration. These free cellæform nuclei were not much affected by the action of acetic acid or water. Lastly, there were seen here and there among the other corpuscles the empty shell of a nucleated cell in the coloured stage, with a free cellæform nucleus beside it as if just extruded by the bursting of the cell-wall. This is a circumstance which may be viewed as additional evidence, and this of a direct kind, in support of the view above given of the nature and origin of the 'red corpuscle' of Man and the Mammifera.—*L. c.*, p. 82.

The author concludes, from his researches, that "the corpuscle of the lymph of vertebrate animals is identical with the corpuscle of their blood; in the oviparous vertebrata, it occurs, like the corpuscle of their blood, in the two phases of granule and nucleated cell; whilst, in man and the mammifera, it occurs, like the corpuscle of their blood, in the three phases of granule-cell, nucleated cell, and free cellæform nucleus." It is further remarked, and the observation is one of much physiological interest, that the fluid of the thoracic duct of a mammiferous animal may, in consequence of the relatively great number of nucleated cells and of the small number of free cellæform nuclei it contains, be compared with the blood of the same animal in its embryo state.—(*L. c.*)

It will not escape the reader how closely these observations bear upon the theory of Hewson. There are doubtless some discrepancies between that theory and the account of Mr. W. Jones, which that gentleman has pointed out; one disagreement being that, whereas Hewson concluded the corpuscles he detected in the juice of the lymphatic glands were merely 'central particles,' the author we are quoting has ascertained that they are in reality nucleated cells, having very distinct nuclei, and extremely pale cell-walls: "in consequence of this, Hewson appears to have overlooked the cell-wall and distinguished the nucleus alone, but when the cell-wall had already become evident around the nucleus by having acquired colour, he naturally supposed that it was a new formation."—(*L. c.* p. 83.) But, notwithstanding this and some other discrepancies, we regard these researches as confirmatory of that which is the fundamental part of Hewson's great theory, namely, that the red corpuscles of the blood are formed or manufactured in the lymphatic system. If this become an established principle in physiology, as we ourselves believe it will be, *provided the lacteal apparatus be included in the term lymphatic system*, then may the patient reiterated investigations of our distinguished countryman, Hewson, and the great truth they will have mainly contributed to elicit, be regarded as one of the most splendid additions the science of organization has ever received.

Before dismissing the consideration of the red corpuscles, we must solicit the attention of our readers to Hewson's experiments on the curious changes these bodies undergo when placed in water and saline solutions. In the following experiment the influence of water in distending the corpuscles, and in making them spherical, whatever may have been their previous form, is described.

"Take a drop of the blood of an animal that has large particles, as a frog, a fish, or what is still better, of a toad; put this blood on a thin piece of glass, as used in the former experiment, and add to it some water, first one drop, then a second, and a third, and so on, gradually increasing the quantity; and in proportion as water is added, the figure of the particle will be changed from a flat to a spherical shape. When much water is added, the vesicle will by degrees become thinner and more transparent, and will at last be dissolved."—*Hewson*, p. 222.

The latter part of this account contains an error, for the red corpuscles are not actually dissolved by water; they are thereby only deprived of their colouring matter, a fact first observed by Dr. Young. The effects produced by saline solutions are thus accurately noticed:—

"If a saturated solution of any of the common neutral salts be mixed with fresh blood, and the globules (as they have been called, but which, for the future, I shall call flat vesicles) be then examined in a microscope, the salt will be found to have contracted or shrivelled the vesicles, so that they appear quite solid, the vesicular substance being closely applied all round the central piece. In proportion as the solution of salt is diluted with water, it has less effect, and when diluted with six, eight, ten or twelve times its quantity of water, it produces no change in the figure of the vesicles, whose flat shape can then be seen, even more distinctly than when mixed with the serum itself."—*L. c.*, p. 229.

A little consideration is sufficient to show that these experiments, which by their delicacy rival some of the more minute manipulations of chemistry, contain the germ of the important discovery subsequently made by Dutrochet as to the absorbing powers of organic membrane; and they also afford the best proof that can be adduced to prove the correctness of the doctrine now universally admitted, that the blood-corpuscles are in fact floating cells. It is true that Hewson himself did not draw any such brilliant inferences from his precise and often-repeated researches; what he might have done, if his life had been longer spared, it would be now vain to speculate. But although these great results escaped him, this profound physiologist firmly established a principle in the physiology of the blood, of which the importance is only now imperfectly comprehended: we allude to the fact that the normal figure of the red corpuscles is preserved by the serum, or, to speak more accurately, by the salts held in solution in that fluid. In answer to the question—Whence is it that the serum has the property of preserving the flat form of the blood-discs? Hewson replies, "it is principally by the salts of the serum that this effect is produced, as is proved by adding a small quantity of any neutral salt to water, when the water is no longer capable of dissolving those particles, nor does it alter their shape when the salt is used in a certain proportion." P. 229.

All these remarks show how powerfully the red corpuscles must be affected, and especially in disease, by the qualities of the liquid vehicle in which they are habitually placed; not merely as regards the influence exerted upon their colour by the salts of the serum, but likewise with respect to their form, and those other physical properties, by which their attraction for each other is, doubtless, greatly modified.

Our notice of these "Experimental Inquiries" has already extended so far, that we have only space for one or two passing allusions to Hewson's description of the Lymphatic System, which, although containing, mixed with the prevailing errors of the day, a large amount of valuable matter, has lost much of its interest in consequence of the more accurate knowledge now possessed respecting the process of absorption. In the following account, the polygonal depressions or cells described by Dr. Sprott Boyd as containing the gastric secreting tubules, and likewise the membranous partitions separating the cells from each other, spoken of by Krause, Huschke and other anatomists as plicæ villosæ, and forming a kind of transition to the true villi of the small intestine, are distinctly noticed.

"At the upper part of the stomach the villous coat appears in a microscope like a honeycomb, or like the reticulum, or second stomach of a ruminant quadruped in miniature; that is, full of small cells, which have thin membranous partitions. Towards the pylorus these partitions are lengthened so as to approach to the shape of the villi of the jejunum." P. 188.

Hewson also describes the honey-combed appearance of the mucous coat of the large intestine; and in the appended extract he has given a clear account of the villi of fishes and reptiles, animals in which, by Rudolphi and others, their existence has been denied: the reader will also mark the network of lacteals here spoken of, and subsequently depicted by Krause. "That the villi in some fish have a network of lacteals, I have distinctly seen in the turbot, where I have injected the lacteals with mercury, which readily runs from those vessels into the villi, and makes them turgid and erected. In the same way I have likewise seen a network of lacteals on the villi of a turtle, where these villi are of a different shape, and in some parts of the gut are cellular, or honeycombed, something like the lower part of the human stomach, only the partitions of the cells are here much larger." And again, in combating the notion of Liberkühn, that each villus has an ampullula, the author says, "of the fallacy of this opinion I was first persuaded from observations made on fish, birds, and amphibious animals, in all of which I can demonstrate that the villi have a network of lacteals as well as a network of arteries and veins."

The account of the compound character of the papillæ of the tongue, anticipates, in some degree, the very accurate investigations of these parts, lately published in the Physiological Anatomy of Dr. Todd and Mr. Bowman.

"The papillæ of the tongue in the human subject appear to the naked eye, when they are not minutely injected, quite smooth, but on a minute injection each of these papillæ appears covered with small vascular processes or villi; so that in such a tongue every one of the papillæ seems in the microscope like a bunch of fibres, or rather like a sheaf of corn; some preparations of which, adapted to the microscope, I have now by me. The learned Albinus seems not only to have observed this, but to have had the same idea of the use of these processes, which he calls tubercles, and has painted them like those little eminences that appear upon a nipple, but I find them much longer."—*L. c.*, p. 193.

An ingenious, and probably the true, explanation of the great number of valves in the absorbent vessels, is assigned in the following passage:—

"The lymphatic system is very full of these valves, much more so than the venous, and the reason of this difference seems to be, that the blood in the venous system is strongly pressed forwards by the vis a tergo from the action of the heart and arteries, and therefore its course is less liable to be interrupted by any accidental pressure. But the motion of the absorbed fluid in these vessels having no such force, but only that of the attraction at the orifice, and the peristaltic contraction of the coats, might easily be overcome by any lateral compression, were it not for the valves, which seem to be given to prevent the retrocession of the lymph being considerable, and to make any lateral pressure, instead of preventing, rather promote its passage to the heart."—*L. c.*, p. 195.

It is interesting to see in what light Hewson regarded that intricate branch of minute anatomy—the structure of the secreting glands. Only a few incidental notices are given; but, from the views expressed in the subjoined extract, so accurate when contrasted with the received opinions of the day, it is evident that he was advancing in the right direction, although still biassed by the erroneous doctrines of Ruysch. In alluding to the difficulty of distinguishing clusters of small vessels, such as those of the Malpighian bodies of the kidneys, from glandular follicles, and to the errors consequent thereon, Hewson remarks:—

"On making a variety of experiments on these other glands, (namely, the mammary and the salivary), I think it evident in what manner the deception has happened to those ingenious anatomists; namely, when the excretory ducts of the breast, for example, are injected with vermillion and painter's size, the small acini of which that gland consists are made extremely red, and such a preparation being dried the acini appear as large as pins' heads, so that the breast has been suspected to have follicles of that magnitude; but on injecting the breast with mercury, which is a brighter substance, and better contrasted to the dried fibres, I have distinguished what in the other preparation might be mistaken for a bag was here evidently no more than the extremity of the excretory duct, terminating in one of these acini, and dividing into a number of branches so suddenly as to come near to Ruysch's description of the penicilli of arteries; but the small branches, into which this extremity of the duct divided, were so close to one another, that in the preparation where they were filled with size and vermillion, they could not be distinguished, but in that where they contained mercury, it evidently appeared that, in each acinus of the magnitude of a pin's head, there was a considerable number of branches, but so small as not easily to be seen with the naked eye."—*L. c.*, p. 191.

In bringing this article to a conclusion, it would be supererogatory to say that these admirable investigations are worthy of universal and careful perusal; the only drawback, indeed, to the satisfaction with which we hail this last production of the Sydenham Society, is the circumstance that such a complete edition of Hewson's works should be confined to the members, numerous as we are happy to know they are, of that institution. To Mr. Gulliver we must again tender our best thanks, for the able manner in which he has discharged the onerous duties devolving on the editor of a series of Inquiries embracing so varied and comprehensive a field as those we have been considering. It was a task requiring no ordinary labour and judgment to illustrate the importance of Hewson's discoveries and observations, by contrasting them with the investigations both of his predecessors and successors; that this great desideratum has been attained in the copious and well-selected notes appended to the present volume, will be, we feel assured, acknowledged by all impartial readers; and with this conviction we congratulate the able editor on the acceptable addition he has made to medical literature.

